# Quality of life, its factors, and sociodemographic characteristics of Polish women with lipedema

**DOI:** 10.1186/s12905-021-01174-y

**Published:** 2021-01-15

**Authors:** Joanna E. Dudek, Wojciech Białaszek, Marcin Gabriel

**Affiliations:** 1 SWPS University of Social Sciences and Humanities, Institute of Cognitive and Behavioral Neuroscience, Chodakowska St. 19/31, 03-815 Warsaw, Poland; 2 University of Medical Sciences, Department of Vascular and Endovascular Surgery, Angiology and Phlebology, Poznań, Poland

**Keywords:** Lipedema, Quality of life, Pain, Physical functioning, Depression

## Abstract

**Background:**

Lipedema is a type of subcutaneous adipose tissue disorder that affects mainly women. Its main symptom is bilateral fat accumulation on the extremities with associated pain in the affected areas. Despite growing interest in lipedema among patients and medical health professionals, lipedema is still often misdiagnosed, misunderstood, and mistreated. To promote better understanding of lipedema, we aimed to investigate factors related to the quality of life and describe selected sociodemographic and clinical characteristics of women with lipedema in Poland.

**Methods:**

We conducted a cross-sectional online survey that was completed by 98 women with lipedema. The participants responded to questionnaires regarding quality of life, sociodemographic and clinical characteristics, and depression symptom severity.

**Results:**

The participants reported low quality of life and high severity of depressive symptoms. The more severe the symptoms related to pain, heaviness, and swelling, the lower the quality of life. Further analyses showed that depression severity mediated this relationship.

**Conclusions:**

The current study provides initial information on screening questions, lipedema symptoms, and comorbidities, pointing to the areas needing in-depth investigation. Further steps to improve quality of life in women with lipedema and to reduce health costs should include the education of medical health professionals, using diagnostic tools that allow for differentiation among diagnoses and precise health risk assessment, and creating Polish treatment guidelines for lipedema.

## Background

Lipedema (also known as *lipoedema)* is a chronic, progressive disorder of adipose tissue that occurs almost exclusively in women [1]. The condition has only recently been included in the International Classification of Diseases (ICD-11) of the World Health Organization as a separate clinical entity in the category “*Certain noninflammatory disorders of subcutaneous fat,*” code EF02.2. According to the ICD-11 description, “*Lipoedema is characterized by non-pitting diffuse ‘fatty’ swelling, usually confined to the legs, thighs, hips and upper arms. It may be confused with lymphoedema*” [2].

The main symptom of lipedema is bilateral subcutaneous fat deposition on the extremities with associated tenderness, pain, and the tendency to bruise easily [1, 3, 4]. Although the condition is still not widely recognized and is often misdiagnosed and mistreated [5], the number of published articles on lipedema has been slowly increasing [6–9]. The etiology of lipedema remains unclear, with a hormonal and genetic component and hypothetical vasculo- and lympho-angiopathy [6, 7, 10, 11]. A number of studies have aimed to identify a diagnostic tool that would allow for the precise diagnosis of lipedema, such as cutaneous ultrasonography [12], computed tomography [13], magnetic resonance [14], lymphoscintigraphy [15], and tissue sodium content [16]. Researchers have also searched for genetic determinants of lipedema in the hope of identifying genetic tests that would allow for the precise differentiation of lipedema from other diagnoses; however, no such determinants have yet been found [17]. Therefore, there is no reliable and easily accessible diagnostic tool or biomarker for lipedema, and diagnosis is based on clinical examination and medical history [6, 7, 18]. The unknown etiology and lack of precise diagnostic tools contribute to a lack of knowledge on the epidemiology of lipedema. Specialists estimate that the disease affects 7 to 11% of adult women in Western countries [5–7, 19]. The prevalence of lipedema in Poland is unknown and has not been previously investigated [18].

A number of studies have indicated that lipedema is related to lower quality of life [9], psychosocial distress, and a number of medical comorbidities and complications [3–7, 20]. Misdiagnosed and untreated lipedema may progress and lead to various physical and mental consequences that generate additional costs for medical healthcare systems, reduced quality of life, disability, and even premature death. These consequences include gait problems and related immobility; secondary lymphedema, which increases the risk of cellulitis and sepsis; venous insufficiency, which may be associated with venous leg ulcers; depression; and weight gain, which might eventually lead to the consequences of associated obesity [3–7, 9, 20].

The existing and accessible treatment of lipedema is focused on symptom reduction and management. Guidelines for lipedema treatment have been created in other countries, including Germany [21], the Netherlands [22], the United Kingdom [23], and the United States of America [24]. They suggest conservative treatment options (manual lymphatic drainage, compression decongestive therapy, physical activity, weight management, lifestyle changes, psychological support) as well as liposuction [3–7, 21–24]. Each of the guidelines emphasizes the importance of self-management as part of a comprehensive and effective treatment of lipedema [9, 20–23, 25].

Lipedema, first described in the 1940s, has been intensely investigated only recently (in the last 10–15 years) [1, 3–7]. In Poland, it has been acknowledged in the scientific world relatively recently, with recognition by the medical community at the 1st Conference of the Lymphological Section of the Polish Phlebological Society in Wrocław in 2016 [26], and further exploration at the 6th Aesthetic Phlebology Conference: Lipoedema—what is the recommended management? in Warsaw in 2019 [27].

In the meantime, following Western countries such as the USA, Australia, the Netherlands, the UK, and Germany, owing to online sources, women have started to organize and form patient support groups. The increased interest in the condition has been reflected by the number of articles on lipedema in the popular press and other media. However, the prevalence of lipedema, patients’ quality of life, clinical characteristics, comorbidities, and psychological functioning in Poland have not previously been scientifically investigated [18]. This knowledge is needed to advance the process of scientific investigation of the condition, draw attention to lipedema and increase evidence to confirm the importance of the condition, its impact on the quality of life and inform guidelines for evidence-based care. In this study, for the first time, we sought to evaluate and describe the sociodemographic and clinical characteristics of Polish women with lipedema and to investigate factors related to their quality of life.

## Methods

We conducted an online survey from March to June 2018. Participants were recruited via a link provided in social media (a Facebook group for Polish women with lipedema, with approximately 500 women in the group at the time of the study). Participation in this study was entirely voluntary, and no incentives were offered to the participants. The study was approved by the Ethics Committee, Faculty of Psychology, SWPS University of Social Sciences and Humanities on the 13th of March 2018 (approval number 7/2018). The survey consisted of an informed consent form, demographic information form, and a set of questionnaires regarding lipedema symptoms, quality of life, depression severity, and psychological aspects of functioning that are beyond the scope of the current study. Quality of life was measured with the Polish version of the World Health Organization Quality of Life (WHOQOL-BREF) scale that assesses four domains: physical health, psychological health, social relationships, and environment. The scale also includes two individually scored items about an individual’s overall perception of quality of life and health. Higher scores indicate higher quality of life [28, 29]. Severity of depression was measured with the Polish adaptation of the Patient Health Questionnaire-9 (PHQ-9) [30]. Participants were asked to rate the frequency of each symptom on a 4-point Likert scale (0 = not at all; 4 = nearly every day) for the previous 28 days. A higher score corresponds to a higher severity of depression. The PHQ-9 has sufficient validity and reliability [30, 31].

In addition, the participants were asked about their weight and height, waist size, and hip circumference to calculate their body mass index (BMI) and waist-to-hip ratio (WHR).

Overall, 130 women participated in the survey. The reported analyses were performed only on surveys that were completed in their entirety (N = 98).

## Results

### Sociodemographic and clinical characteristics

The age of the participants ranged from 22 to 73 years (M = 40.43, SD = 10.43). Of the participants, 85 (86.73%) reported living in an urban area, and 57 (58.2%) had sedentary work. Most participants declared that they estimate their economic status as good (n = 41, 41.3%) or average (n = 43, 43.9%). Approximately half of the participants (n = 51, 52%) reported having a higher education degree.

Almost half of the participants reported having been diagnosed with lipedema by a medical health practitioner (n = 44, 44.9%). In the current study, we found no significant differences between diagnosed versus non-diagnosed women in any of the variables, including lipedema symptom severity; therefore, we present analyses for the whole sample.

Online sources such as website articles and social media were the most common initial sources of knowledge of lipedema (n = 61, 62.3%). However, almost one-fifth of the participants had initially heard of lipedema from a health professional (n = 17, 17.3%). Other sources of information included magazines, family members, TV shows, and friends.

Most women estimated that their first symptoms of lipedema had occurred during puberty (n = 43, 43.9%) or childhood (n = 13, 13.3%). The majority of the participants had not undergone liposuction (88.8%, n = 87).

Using the data provided by the participants, we calculated their BMI and WHR. The average BMI was 30.8 (SD = 7.1), and the average WHR was 0.78 (SD = 0.1). Based solely on the BMI score, 75 participants (76.5%) could be classified as overweight (n = 26, 26.5%) or obese (n = 49, 50%), whereas according to the WHR, only 21 (21.42%) could be classified as obese and therefore subject to the types of health risks associated with obesity [32, 33]. The self-reported data related to lipedema and the clinical characteristics of the participants are shown in Table 1.

**Table 1:** Self-reported clinical information on the participants

Variable	Value
Age (y) mean (SD)	40.43 (10.43)
Confirmed diagnosis, N (%)	44 (44.9)
Initial source of information on lipedema, N (%)	
Online	61 (62.3)
Health professional	17 (17.3)
Other	20 (20.4)
Lipedema onset, N (%)	
Childhood	13 (13.3)
Puberty	43 (43.9)
Pregnancy	11 (11.2)
Menopause	5 (5.1)
Other	13 (13.3)
Hard to tell	5 (5.1)
Liposuction, N(%)	11 (11.2)
BMI^a^, N (%)	
18.5–24.99 (normal weight)	20 (20.4)
25–29.99 (overweight)	26 (26.5)
30–34.99 (obesity class I)	29 (29.6)
35–39.99 (obesity class II)	11 (11.2)
≥ 40 (obesity class III)	9 (9.2)
Missing data	3 (3.1)
WHR, N (%)	
< 0.85	62 (63.3)
≥ 0.85	21 (21.4)
Missing data	15 (15.3)

^a^World Health Organization. Physical status: the use and interpretation of anthropometry. Report of a WHO Expert Committee. World Health Organ Tech Rep Ser. 1995; 854, 1- 452

Physical activity, which is an important part of self-management and an important aspect of health-related outcomes in women with lipedema, was also measured in the current study. Most of the women reported one to three hours of physical activity weekly (n = 34, 34.6%) or more (n = 27, 27.6%). Surprisingly, only 2% of the participants reported swimming or aquatic exercise as a common activity despite the importance of these activities in managing lipedema (Table 2).

**Table 2:** Level and type of physical activity and comorbidities in women with lipedema

Variable	Value
Level of physical activity (weekly), N (%)	
More than 3 h weekly	27 (27.6)
1 to 3 h weekly	34 (34.6)
Less than 1 h weekly	29 (29.6)
No	8 (8.2)
Type of physical activity, N (%)	
Walking	46 (46.9)
Cycling	9 (9.2)
Aerobic exercise	5 (5.1)
Gym	8 (8.2)
Running	4 (4.1)
Yoga	2 (2)
Aquatic exercise/swimming	3 (3)
Other	16 (16.3)
No	5 (5.1)
Comorbidities (self-reported), N (%)	
Hypothyroidism	31 (31.6)
Lymphedema	30 (30.6)
Venous insufficiency	20 (20.4)
Arthritis	20 (20.4)
Insulin resistance	13 (13.3)
Hypertension	4 (4.1)
Polycystic ovary syndrome	5 (5.1)
Irritable bowel syndrome	4 (4.1)
Fibromyalgia	4 (4.1)
None	18 (18.4)

We asked about a number of other chronic conditions that affect women with lipedema. The most common comorbidities reported were hypothyroidism (n = 31, 31.6%), lymphedema (n = 30, 30.6%), venous insufficiency (n = 20, 20.4%), and arthritis (n = 20, 20.4%). Only 18 participants (18.4%) reported having no comorbidity (Table 2).

### Lipedema symptoms

**Fig. 1:**
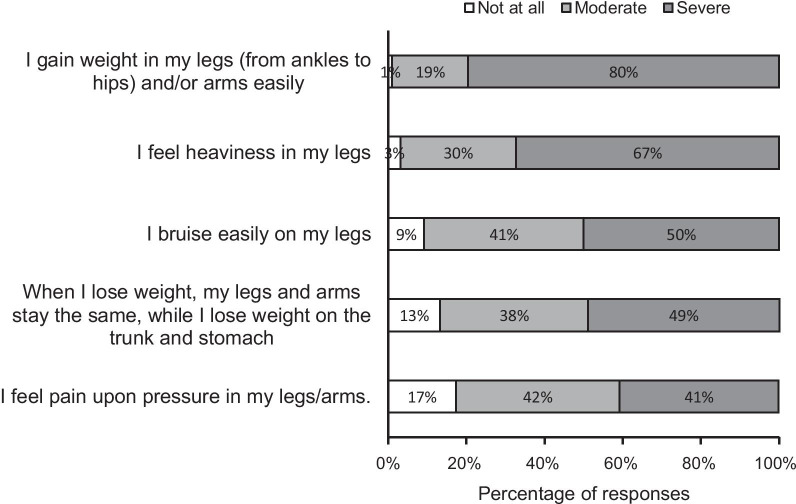
Participants’ ratings of intensity of symptoms based on lipedema symptoms criteria

To check whether the participants met the criteria of the common screening tool used for lipedema by patient organizations [34], we asked participants to what extent they had experienced each of the lipedema symptoms (criteria). Most of the participants reported moderate to severe intensity of easily gaining weight in the legs/arms (n = 97, 99%), feelings of heaviness in their legs (n = 95, 96.9%), easy bruising (n = 89, 90.8%), difficulty losing weight from their legs/arms (n = 85, 86.8%), and pain upon pressure (n = 81, 82.6%). The majority of the participants (n = 67, 68.4%) had experienced all five symptoms; 22.4% (n = 22), four symptoms; 7.1% (n = 7), 3 symptoms; 1% (n = 1), two symptoms; and 1%, only one of the 5 symptoms (Fig. 1).

**Fig. 2:**
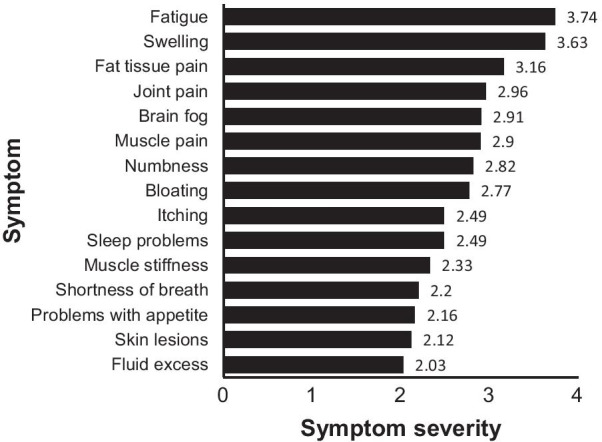
Participants’ mean scores for severity of lipedema symptoms

Furthermore, we assessed lipedema symptom severity by asking about various symptoms based on the questionnaire used in clinical practice for patients with lipedema by Karen Herbst [35] and in previous research [36]. The participants were asked to rate the severity of lipedema symptoms in the previous 28 days (e.g., fat tissue pain, swelling) using a 5­point Likert scale (0 = no problem, 4 = extremely severe). An overall sum of the aforementioned symptoms was used as a measure of lipedema symptom severity. The data are illustrated in Fig. 2.

The symptoms with the highest mean severity were leg heaviness (M = 3.96, SD = 1.03), fatigue (M = 3.74, SD = 0.97), swelling (M = 3.63, SD = 1.08), and fat tissue pain (M = 3.13, SD = 1.23). Most of the participants assessed the severity of leg heaviness in the previous 28 days as moderate to extremely severe (n = 91, 92.9%), and only 3 participants had not experienced that symptom at all. Similarly, almost all participants reported moderate to severe fatigue (n = 91, 92.9%), swelling (n = 86, 87.8%) and fat tissue pain (n = 71, 72.4%).

### Quality of life, depression severity, and lipedema symptoms

More than one-third of the participants assessed their overall quality of life as good or very good (n = 34, 34.7%), while 20 (20.4%) rated their quality of life as poor or very poor (M = 3.12, SD = 0.85). The degree of lipedema patients’ satisfaction with their own health was assessed as low by more than half of the participants, and 59 reported being dissatisfied or very dissatisfied (60.1%). The average rating was 2.36 (SD = 0.92). The transformed mean scores for the domains of the WHOQOL-BREF were 45.4 (SD = 16.9) for physical health, 46.3 (SD = 17.5) for psychological health, 50.4 (SD = 20.8) for social relationships, and 49.6 (SD = 14.2) for environment.

The average total score for the PHQ-9 (assessing depression severity) was 12.2 (SD = 6.07). According to the score categories for the PHQ-9 (5–9 = mild, 10–14 = moderate, 15–19 = moderately severe, and 20 or higher = severe), more than half of the participants (n = 56, 59.2%) reported a level of severity indicating that they may be suffering from depression, and 11% reported a level indicating severe depression (Table 3).

**Table 3:** Depression symptom severity in 98 women with lipedema

PHQ-9 score	n	%
≤ 4 (minimal depression)	9	9.2
5–9 (mild depression)	31	31.6
10–14 (moderate depression)	27	27.6
15–19 (moderately severe depression)	20	20.4
≥ 20 (severe depression)	11	11.2

To show how various lipedema symptoms contribute to quality of life and depression severity, first we conducted exploratory factor analysis on sixteen discussed symptoms. This step was done to simplify the structure of symptoms and test whether reduction to more general categories was possible. First, to select the best factorial solution, we conducted Velicer’s MAP (minimum average partial; [37]) test on the ratings of sixteen symptoms, which achieved minimum (min = 0.034) with three factor solution. Second, we utilized exploratory factor analysis (principal axis factoring with oblimin rotation; Keiser–Meier–Olkin criterion of 0.869). We found that the first factor explained 38.8% of the variance, the second factor explained 7.5%, and the third factor explained 6.4% of the variance. The pattern matrix indicated no severe cross-loadings except for fatigue, which loaded on factors 1 and 2 (see Table 4) . We decided to retain this symptom in further analyses. To simplify the structure of symptom severity and test their role in quality of life, we computed simple unweighted average scores on each of the factors and used these indicators as predictors in a multiple regression analysis. Factor 1 consisted of the symptoms associated with the main features of lipedema such as leg heaviness, fat tissue pain, swelling, joint and muscle pain, as well as muscle stiffness. Factor 2 included more general symptoms such as problems with appetite, sleeping, brain fog, fatigue, and bloating. Factor 3 consisted of other symptoms (itching, skin lesions, fluid excess, shortness of breath, and numbness). The model with three predictor variables and quality of life as the dependent variable explained a significant 23.5% of the variance (F(3, 94) = 10.95, *p* < 0.001). Importantly, factor 1 was a significant predictor of quality of life (t(94) = 2.921, *p* = 0.004; β = − 0.345), and factors 2 and 3 were not significant (factor 2: t(94) = 1.631, *p* = 0.106; β = -0.176; factor 3: t(94) = 0.596, *p* = 0.552; β = − 0.071). These results indicated the higher the severity of the symptoms associated with the main features of lipedema, such as pain, heaviness, and swelling, the lower the quality of life.

**Table 4:** Factor loadings for 16 symptom severity scores from pattern matrix across three factors

	Factor 1	Factor 2	Factor 3
Leg heaviness	**0.896**	− 0.061	0.08
Joint pain	**0.63**	− 0.039	− 0.022
Fat tissue pain	**0.613**	− 0.004	− 0.130
Muscle pain	**0.604**	0.176	− 0.037
Swelling	**0.573**	− 0.034	− 0.294
Muscle stiffness	**0.449**	0.241	− 0.304
Brain fog	0.069	**0.855**	0.147
Problems with appetite	− 0.153	**0.560**	− 0.120
Bloating	0.08	**0.541**	− 0.125
Fatigue	0.471	**0.472**	0.081
Sleep problems	0.123	**0.370**	− 0.201
Itching	− 0.043	− 0.002	− **0.835**
Skin lesions	0.033	− 0.067	− **0.737**
Shortness of breath	0.142	0.098	− **0.678**
Fluid excess	− 0.022	0.277	− **0.607**
Numbness	0.302	0.001	− **0.496**

Factor loadings corresponding to a given factor appear in bold

So far in this section we demonstrated the role of various groups of symptoms on quality of life. To test what might be a possible mechanism, or the process by which factor 1 impacts quality of life, we decided to test the mediating role of depression severity. The model with factor 1 symptoms as the independent variable, depression as the mediating variable, and quality of life as a dependent variable was statistically significant, explaining 53.2% of the variance (F(2, 95) = 54.831, *p* < 0.001). Using 5000 bootstrapped samples (95% confidence intervals) [38], we found that the indirect effect of depression severity on the relation between factor 1 symptoms and quality of life was statistically significant as indicated by the 95% bootstrapped interval (CI [− 9.668, − 3.999], a*b = 3.834* − 1.632 = − 6.256). The direct effect (c′ = − 1.951) was not significant, as indicated by the confidence interval (CI [− 4.824, 0.923]). These results indicate that in this sample of women with lipedema, symptom severity (i.e., factor 1 symptoms related to pain, heaviness, and swelling) was positively associated with depression severity, which thereby decreased their quality of life.

## Discussion

We investigated the sociodemographic and clinical characteristics of Polish women with lipedema, their quality of life and its factors. This is the first study to focus on Polish women with this condition.

In the current study, the participants reported that the most severe symptoms were leg heaviness, fatigue, swelling and fat tissue pain, which is in line with previous studies [39–41]. More than half of the participants (59.2%) reported a heightened level of depressive symptoms, which also has been observed and reported in other studies [20, 36, 40]. The quality of life of the participants, measured by the WHOQOL-BREF, was lower in all domains (physical health, psychological health, social relationships, and environment) than in the general population [42]. The obtained results are in accordance with a number of previous studies examining quality of life in women with lipedema in international samples [9, 20, 39]. In our study, quality of life was predicted by the severity of symptoms related to pain, heaviness, and swelling. Further analyses showed that depression severity mediated the relationship between symptom severity and quality of life. The higher the severity of symptoms such as fat tissue pain, leg heaviness, muscle stiffness, swelling, and muscle and joint pain, the higher the severity of depression, which leads to lower quality of life. This result points out a need to focus not only on physical but also psychological complaints when assessing the functioning of women with lipedema [5–7, 41]. One of the possible explanations for the relationships between quality of life, depression, and symptom severity in women with lipedema in Poland is there continues to be little knowledge of the condition among medical health professionals and the general public as well as a lack of guidelines and low access to reliable information in the Polish language, resources, and treatment. Women struggling with symptoms related to leg heaviness, pain, and swelling may have experienced not only stigma and mistreatment, but also failure in their attempts of misunderstood self-management which could put them at risk of developing depression. For example, although the women in the current study reported being engaged in one or more hours of physical activity per week, only 2% mentioned aquatic exercise as the most commonly chosen activity, even though all the guidelines agree that aquatic exercise is the most beneficial activity for women with lipedema [21–23]. An explanation of this finding, beyond a lack of knowledge on the possible benefits of aquatic exercise, might be the high cost of such an activity; difficulty in accessing facilities that offer such training; and psychological reasons such as depression, appearance-related shame, and anxiety.

Simultaneously, we acknowledge that interpretation of the relationship between quality of life, depression, and symptom severity is limited by the cross-sectional design. We emphasize that, similar to a study by Dudek et al. [36], the relationship observed in the current study is correlational and has never been seen as causal as suggested by Bertsch and Erbacher [43]. It cannot be excluded that being depressed may affect how one responds and perceives their symptoms or quality of life. There is also a possibility that the observed relationship between depression severity and lipedema symptom severity may result from underlying inflammatory processes [44, 45], from the associated obesity [46], or from other factors that the study did not take into account. Further research should focus on investigating causal relationships between variables using longitudinal or experimental designs. Nevertheless, taking into account depression severity is important for quality of life as well as for the effectiveness of treatments, as patients may be stuck in a vicious cycle of depression. The lowered mood and lack of engagement in vital activities specific to depression may be obstacles to engaging in self-management procedures, healthy behaviors, and physical activities, and may impact compliance.

This study is the first to acknowledge and emphasize that lipedema affects women in Poland. In our online, voluntary survey, almost half of the participants reported having been diagnosed by medical health professionals (44.9%). Almost one-fifth, 17.3%, had initially learned about lipedema from medical health professionals. These results emphasize the importance of knowledge of lipedema among medical health professionals and highlight the importance of the role that professionals play in the diagnosis and treatment of the illness. Thus far, lipedema has not been widely known among professionals in Western countries [5–7, 47]. A study on specialists from the Vascular Society of Great Britain and Ireland showed that lipedema was recognized by only 46.2% [48]. To the best of our knowledge, the level of knowledge in the Polish medical community has not yet been measured.

More than half of the surveyed women (57.1%) reported first noticing the onset of symptoms of the disease at puberty or even during childhood, which is similar to the results obtained in other studies [5, 6, 39, 40]. For instance, in a study of Dutch women with lipedema, 64.2% of the participants reported the onset of lipedema at the age of 10 to 19 years [40].

Half of the participants had a BMI score that could be classified as obese, which is a result similar to that observed in other studies [5, 6, 39, 41]. However, according to the WHR, only 21.2% of the women were classified as obese [33]. This result emphasizes the importance of gathering a number of different measurements in screening for health risks associated with obesity, such as hypertension, diabetes mellitus, heart disease, and stroke, when treating at-risk patients. The obtained results support the existing doubts about the relevance of BMI as a useful screening tool in the assessment of obesity among women with lipedema [5–7, 39, 49].

The importance of appropriate health risk assessment tools is further emphasized by the results regarding comorbidities. In our study, the most frequently reported comorbidities were lymphedema, arthritis, hypothyroidism, and venous insufficiency. Thirty percent of the participants reported having hypothyroidism, in comparison to 11.7% in previously-mentioned study of Dutch women [40], but only 4% reported having hypertension, in comparison to 18.4% in the Dutch study. Typical obesity-associated comorbidities were not reported by the participants, which is in line with the current state of knowledge of lipedema-associated health risks [6, 20, 41]. In addition, in the current study, we did not observe a heightened frequency of fibromyalgia (only 4%) or hypermobility, both of which have been reported to co-occur with lipedema [5–7, 20, 41]. For instance, in the study by Beltran and Herbst [41] comparing patients with Dercum’s disease and with lipedema, hypermobility was more common among the latter. Of 160 patients with lipedema in that study, more than half (58%) had been diagnosed with hypermobility, which was assessed with the Beighton score. Thus, the lack of reported incidence of hypermobility in the current study may be explained by the lack of proper methods of assessment or the rare occurrence of the condition as well as the lack of awareness of this particular comorbidity in Poland [50].

Most of the participants in our study (68.4%) reported five symptoms, and 22.4% reported four symptoms used in screening tests for lipedema. From all the symptoms being investigated we identified three sets of symptoms: (1) symptoms related to pain, swelling, and heaviness; (2) general symptoms (e.g., brain fog, fatigue); and (3) other symptoms (e.g., skin lesions, fluid excess, numbness). In particular, the set of symptoms related to pain, swelling, and heaviness predicted quality of life. It might be useful to create a brief questionnaire that includes assessment of the patient’s symptoms related to pain, swelling, and heaviness, which can be used in clinical practice as a screening tool to identify women at risk and refer them for further diagnosis. Early and precise diagnosis—as precise as possible with the current diagnostic tools—will allow practitioners to differentiate lipedema from obesity, Dercum’s Disease, and lymphedema, enabling them to provide disease-specific, cost-effective treatment and prevention of disease-specific risks [5–7, 20]. Knowledge of the diagnosis and offering disease-specific treatment may not only improve the long-term health status, quality of life, and well-being of women affected by lipedema but also improve patient-provider cooperation, especially in the area of self-management. For example, a low-caloric diet may not lead to weight loss in women with lipedema [1, 5–7, 20]. However, weight management and a healthy, anti-inflammatory diet may improve overall health and slow the progression of lipedema, decreasing the risk of complications [21–23]. Another example is physical activity. Intense aerobic exercise without compression garments may be counterproductive for women with lipedema; however, exercising in compression garments, especially aquatic exercise, and moderate exercise may be very beneficial, reducing pain and swelling. Having realistic expectations and understanding their condition may help patients to engage in and sustain healthy behaviors and slow the progression of the illness [21–23, 25]. Moreover, it may improve their sense of self-efficacy and decrease psychological distress.

The current study is the first to explore quality of life in women with lipedema in Poland, and it has a number of limitations. The study was conducted online and is based on self-reported data. Participation in the study was voluntary, and a link was provided in an online social media support group, which might have created selection bias. The women who participated in the study might have been either more dissatisfied with the condition than average or overly motivated and interested in the topic. The women using the social media group for support may be more active and creative in searching for solutions and may use social support more effectively than other women with lipedema. Despite the enormous advantages and progress that online sources have brought to lipedema, they may also be a problem, leading to overestimation of the prevalence of lipedema [51] and the risks related to self-diagnosis, and adding even more confusion regarding diagnosis and lipedema treatment.

In addition, because all collected data were through self-report, to some extent participants might have been biased or inaccurate in their responses. In particular, inaccuracy might exist in the self-reported measurements used to calculate BMI and WHR. In further studies, such measurements should be performed by trained professionals (e.g., nurses or physiotherapists). However, the current study is the first to investigate aspects of living with lipedema among Polish women, and given there is no active association for Polish patients, using an online support group to recruit participants for this study was the only available option.

## Conclusions

The purpose of the current study was to increase knowledge of the clinical characteristics, of women with lipedema in Poland, and their quality of life and its factors. Additionally, through this investigation our aim was to identify further directions for research and possible interventions. The results indicated the higher the severity of symptoms related to pain, heaviness, and swelling the lower the quality of life, and that depression severity mediated this relationship. Therefore, symptom management (preventing pain and swelling) and addressing psychological functioning may play a role in improving quality of life in women with lipedema. Furthermore, there is a need for proper knowledge, diagnosis, and treatment of lipedema in Poland. Increased knowledge on the condition will allow for further studies and effective treatment (with additional focus given to self-management). Researchers should investigate the prevalence of the condition, deepening the knowledge of the symptoms, comorbidities (both physical and psychological), and the course of the disease. Such research will lead to the development of appropriate screening tools to assess women at risk and provide them with an opportunity to obtain early diagnosis. There is also a need to describe treatment options in Polish, develop lipedema guidelines for practitioners and patients to prevent progression and complications of the condition, decrease health care costs, and improve women’s quality of life and well-being.

## Data Availability

The datasets used and/or analyzed during the current study are available from the corresponding author on reasonable request.

## Abbreviations

BMI: Body mass index
WHR: Waist-to-hip ratio

## References

[1] Wold LE, Hines EA, Allen EV (1951). Lipedema of the legs; a syndrome characterized by fat legs and edema. Ann Intern Med.

[2] World Health Organization. International statistical classification of diseases and related health problems (11th Revision) http://id.who.int/icd/entity/1172950828 Accessed 1 July 2019.

[3] Herbst KL (2012). Rare adipose disorders (RADs) masquerading as obesity. Acta Pharmacol Sin.

[4] Forner-Cordero I, Szolnoky G, Forner-Cordero A, Kemény L (2012). Lipedema: an overview of its clinical manifestations, diagnosis and treatment of the disproportional fatty deposition syndrome - systematic review. Clin Obes.

[5] Buck DW, Herbst KL (2016). Lipedema: a relatively common disease with extremely common misconceptions. Plast Reconstr Surg Glob Open.

[6] Wollina U (2019). Lipedema—an update. Dermatol Ther.

[7] Tuğral A, Bakar Y (2019). An approach to lipedema: a literature review of current knowledge of an underestimated health problem. Eur J Plast Surg.

[8] Redondo Galán C, García Bascones M, Marquina Valero MA (2019). Lipedema: clínica, diagnóstico y tratamiento. Revisión de la literature Rehabilitación.

[9] Alwardat N, Di Renzo L, Alwardat M, Romano L, De Santis GL, Gualtieri P (2019). The effect of lipedema on health-related quality of life and psychological status: a narrative review of the literature. Eat Weight Disord.

[10] Szél E, Kemény L, Groma G, Szolnoky G (2014). Pathophysiological dilemmas of lipedema. Med Hypotheses.

[11] Al-Ghadban S, Cromer W, Allen M, Ussery C, Badowski M, Harris D (2019). Dilated blood and lymphatic microvessels, angiogenesis, increased macrophages, and adipocyte hypertrophy in lipedema thigh skin and fat tissue. J Obes.

[12] Iker E, Mayfield CK, Gould DJ, Patel KM (2019). Characterizing lower extremity lymphedema and lipedema with cutaneous ultrasonography and an objective computer-assisted measurement of dermal echogenicity. Lymphat Res Biol.

[13] Monnin-Delhom ED, Gallix BP, Achard C, Bruel JM, Janbon C (2002). High resolution unenhanced computed tomography in patients with swollen legs. Lymphology.

[14] Lohrmann C, Foeldi E, Langer M (2009). MR imaging of the lymphatic system in patients with lipedema and lipo-lymphedema. Microvasc Res.

[15] Forner-Cordero I, Oliván-Sasot P, Ruiz-Llorca C, Muñoz-Langa J (2018). Lymphoscintigraphic findings in patients with lipedema. Rev Esp Med Nucl Imagen Mol.

[16] Crescenzi R, Marton A, Donahue PMC, Mahany HB, Lants SK, Wang P (2018). Tissue sodium content is elevated in the skin and subcutaneous adipose tissue in women with lipedema. Obesity.

[17] Paolacci S, Precone V, Acquaviva F, Chiurazzi P, Fulcheri E, Pinelli M (2019). Genetics of lipedema: new perspectives on genetic research and molecular diagnoses. GeneOb Project. Eur Rev Med Pharmacol Sci.

[18] Łyszczak P, Szuba A (2018). Lipedema: a clinical entity. Acta Angiol.

[19] Marshall M, Schwahn-Schreiber C (2011). Prävalenz des Lipödems bei berufstätigen Frauen in Deutschland (Lipödem-3-Studie). Phlébologie.

[20] Torre YS, Wadeea R, Rosas V, Herbst KL (2018). Lipedema: friend and foe. Horm Mol Biol Clin Investig.

[21] Reich-Schupke S, Schmeller W, Brauer WJ, Cornely ME, Faerber G, Ludwig M (2017). S1 guidelines: lipedema. J Dtsch Dermatol Ges.

[22] Halk AB, Damstra RJ (2017). First Dutch guidelines on lipedema using the international classification of functioning, disability and health. Phlebology.

[23] Hardy D, Williams A (2017). Best practice guidelines for the management of lipoedema. Br J Community Nurs.

[24] Herbst KL. Standard of care for Lipedema in the United States. Grant funded by National Heart Lung and Blood Institute (NHLBI). http://app.dimensions.ai/details/grant/grant.8388531 Accessed 1 July 2019.

[25] Fetzer A, Wise C. Living with lipoedema: reviewing different self-management techniques. Br J Community Nurs. 2015; Suppl Chronic:S14, S16–9. 10.12968/bjcn.2015.20.Sup10.S14 26418584

[26] 1st Conference of the Lymphological Section of the Polish Phlebological Society, Wroclaw 22–23.04.2016. Lymphedema—pathophysiology, diagnosis and treatment. Acta Angiol. 2016;22:60–70.

[27] 6th Aesthetic Phelbology Conference. Lipoedema– what is the recommended management? Warsaw 17.11.2019. https://www.termedia.pl/Konferencja-6th-Aesthetic-Phlebology-Conference-Intro,1058,8982.html. Accessed 19 Nov 2019.

[28] Skevington SM, Lotfy M, O'Connell K (2004). The World Health Organization's WHOQOL-BREF quality of life assessment: psychometric properties and results of the international field trial. A report from the WHOQOL group. Qual Life Res..

[29] Jaracz K, Kalfoss M, Górna K, Baczyk G (2006). Quality of life in Polish respondents: psychometric properties of the Polish WHOQOL–Bref. Scand J Caring Sci.

[30] Kroenke K, Spitzer RL, Williams JB (2001). The PHQ-9: validity of a brief depression severity measure. J Gen Intern Med.

[31] Kokoszka A, Jastrzębski A, Obrębski M (2016). Ocena psychometrycznych właściwości polskiej wersji Kwestionariusza Zdrowia Pacjenta-9 dla osób dorosłych [Psychiatry]. Psychiatria.

[32] World Health Organization. Physical status: the use and interpretation of anthropometry. Report of a WHO Expert Committee. World Health Organ Tech Rep Ser. 1995; 854, 1–452. 8594834

[33] World Health Organization: Waist Circumference and Waist-Hip Ratio: Report of a WHO Expert Consultation. Geneva: 2008.

[34] The Lipedema Project. Do you have lipedema? Quiz. http://lipedemaproject.org/do-you-have-lipedema-quiz/ Accessed 4 Jan 2018.

[35] Herbst KL. Symptom tracker. http://lipomadoc.org Accessed 4 Jan 2018.

[36] Dudek JE, Białaszek W, Ostaszewski P, Smidt T (2018). Depression and appearance-related distress in functioning with lipedema. Psychol Health Med.

[37] Velicer WF (1976). Determining the number of components from the matrix of partial correlations. Psychometrika.

[38] Hayes AF (2018). Introduction to mediation, moderation, and conditional process analysis: a regression-based approach.

[39] Dudek JE, Białaszek W, Ostaszewski P (2016). Quality of life in women with lipoedema: a contextual behavioral approach. Qual Life Res.

[40] Romeijn JR, de Rooij MJ, Janssen L, Martens H (2018). Exploration of patient characteristics and quality of life in patients with lipoedema using a survey. Dermatol Ther.

[41] Beltran K, Herbst KL (2017). Differentiating lipedema and Dercum’s disease. Int J Obes.

[42] Hawthorne G, Herrman H, Murphy B (2006). Interpreting the WHOQOL-BREF: preliminary population norms and effect sizes. Soc Indic Res.

[43] Bertsch T, Erbacher G (2018). Lipedema—myths und facts part 1. Phlébologie.

[44] Abdallah CG, Geha P (2017). Chronic pain and chronic stress: two sides of the same coin?. Chronic Stress.

[45] Jaremka LM, Fagundes CP, Glaser R, Bennett JM, Malarkey WB, Kiecolt-Glaser JK (2013). Loneliness predicts pain, depression, and fatigue: understanding the role of immune dysregulation. Psychoneuroendocrinology.

[46] Petroni ML, Villanova N, Avagnina S, Fusco MA, Fatati G, Compare A (2017). Psychological distress in morbid obesity in relation to weight history. Obes Surg.

[47] Shavit E, Wollina U, Alavi A (2018). Lipoedema is not lymphoedema: a review of current literature. Int Wound J.

[48] Tiwari A, Myint F, Hamilton G (2006). Management of lower limb lymphoedema in the United Kingdom. Eur J Vasc Endovasc Surg.

[49] Child AH, Gordon KD, Sharpe P, Brice G, Ostergaard P, Jeffery S (2010). Lipedema: an inherited condition. Am J Med Genet A.

[50] Szalewska M, Lupa M, Pochroń N, Pudzianowski S, Szalewski L, Bożyk A (2014). Prevalence of joint hypermobility syndrome amongst dental students of the Medical University of Lublin. Pol J Public Health.

[51] Reich-Schupke S, Altmeyer P, Stücker M (2013). Thick legs - not always lipedema. J Dtsch Dermatol Ges.

